# Selective neural electrical stimulation restores hand and forearm movements in individuals with complete tetraplegia

**DOI:** 10.1186/s12984-020-00676-4

**Published:** 2020-05-19

**Authors:** Wafa Tigra, Mélissa Dali, Lucie William, Charles Fattal, Anthony Gélis, Jean-Louis Divoux, Bertrand Coulet, Jacques Teissier, David Guiraud, Christine Azevedo Coste

**Affiliations:** 1 INRIA, University of Montpellier, CNRS, Montpellier, France; 2 MXM group, Sophia-Antipolis, France; 3 La Châtaigneraie Rehabilitation Center, Menucourt, France; 4 Propara Rehabilitation Center, Montpellier, France; 5 Axonic, MXM Group, Sophia-Antipolis, France; 6 University Hospital of Montpellier, Montpellier, France; 7 Beau Soleil Clinic, Montpellier, France; 8 NEURINNOV SAS, Montpellier, France

**Keywords:** Neural stimulation, Tetraplegia, Grasping movement, Multicontact cuff electrode, Selective stimulation

## Abstract

**Background:**

We hypothesized that a selective neural electrical stimulation of radial and median nerves enables the activation of functional movements in the paralyzed hand of individuals with tetraplegia. Compared to previous approaches for which up to 12 muscles were targeted through individual muscular stimulations, we focused on minimizing the number of implanted electrodes however providing almost all the needed and useful hand movements for subjects with complete tetraplegia.

**Methods:**

We performed acute experiments during scheduled surgeries of the upper limb with eligible subjects. We scanned a set of multicontact neural stimulation cuff electrode configurations, pre-computed through modeling simulations. We reported the obtained isolated and functional movements that were considered useful for the subject (different grasping movements).

**Results:**

In eight subjects, we demonstrated that selective stimulation based on multicontact cuff electrodes and optimized current spreading over the active contacts provided isolated, compound, functional and strong movements; most importantly 3 out of 4 had isolated fingers or thumb flexion, one patient performed a Key Grip, another one the Power and Hook Grips, and the 2 last all the 3 Grips. Several configurations were needed to target different areas within the nerve to obtain all the envisioned movements. We further confirmed that the upper limb nerves have muscle specific fascicles, which makes it possible to activate isolated movements.

**Conclusions:**

The future goal is to provide patients with functional restoration of object grasping and releasing with a minimally invasive solution: only two cuff electrodes above the elbow.

Ethics Committee / ANSM clearance prior to the beginning of the study (inclusion period 2016–2018): CPP Sud Méditerranée, #ID-RCB:2014-A01752–45, first acceptance 10th of February 2015, amended 12th of January 2016.

**Trial registration:**

(www.clinicaltrials.gov): #NCT03721861, Retrospectively registered on 26th of October 2018.

## Background

The incidence of spinal cord injuries (SCIs) in Western Europe and the United States is estimated at 16 and 40 cases per million, respectively [1], with the proportion of cervical injuries steadily increasing. SCIs can have a devastating impact on patient health, autonomy and quality of life. Technical aids (e.g., motorized wheelchairs, orthoses, medical electric beds, transfer boards, home automation, etc.) can restore some independence to people with tetraplegia, but recovery of gripping movements is still the priority [2–5]. Indeed, most activities of daily living are performed via hand movements and therefore restoration of active forearm, hand and wrist motricity would help patients recover greater autonomy and thus increase their quality of life. In the absence of spinal cord repair, only partial solutions are available today for this population. Functional surgery is mainly based on musculotendinous transfers and has been used to restore partial movements of the hand and wrist for several decades. A set of muscles under voluntary contraction are therefore used, with the only condition being that their initial function is compensated by agonist muscles still under voluntary control [6]. More recently, nerve transfers have been attempted to re-innervate paralyzed muscle to recover voluntary control [7]. However, both methods require a sufficient number of muscles or nerves that are still under voluntary control. The transferred as well as the remaining agonist muscles should be strong enough to ensure efficient recovery [8]. The alternative is to use an implanted or external functional electrical stimulation (FES) device, provided that the sublesional paralyzed muscles are still innervated by intact motoneurons. One of the first applications of FES to recover hand motion was reported by Catton and Backhouse in 1954 [9]. FES was then used to recover grip movements in patients with high tetraplegia as early as 1963 [10–12]. These devices used either intramuscular or epimysial electrodes, requiring one electrode for each muscle involved in the targeted movement. Surface electrode arrays [13] may be used but are clearly limited in their present form to the laboratory. Moreover, external or percutaneous devices are very limited in terms of acceptability, efficiency and benefits and thus are not used by patients in a daily-living context. The only successful device has been the FreeHand system: more than 250 patients [14] have had it successfully implanted with clear benefits [15, 16]. Up to 12 muscular electrodes have been implanted [17], but a research version attempted to replace several muscle electrodes with a single neural 4-contact electrode [18]. The results on selectivity remained limited during the implanted phase, due to the adopted approach based on a monopolar scanning of the available electrode contacts. The same team also tried neural cuff electrodes to provide movements in the whole upper limb but the setup required 2 implants, 12 to 14 intramuscular electrodes and 6 4-contact cuffs electrodes [19]. The implantation needed 3 (resp. 2) surgeries for the first (resp. second) patient. In both cases, one implant was almost dedicated to the fingers, thumb, wrist movements with 12 intramuscular electrodes. The second implant was mostly dedicated to elbow and shoulder movements with 2 intramuscular electrodes and the use of cuff electrodes limited to the best contact response among the 4 against a global reference leading to a monopolar like stimulation that is not the most selective one. Simplified steering current paradigm was also tested and showed increased selectivity even though limited to the radial and musculocutaneous nerves [20]. The FreeHand device was commercialized but was limited by the complexity of the surgery due to the high number of muscle electrodes implanted over the arm/forearm/hand; the surgery was estimated to last 5 h with an access to all targeted muscles [15] and it is not commercialized since 2001.

A neat solution would consist of activating groups of muscles through a limited number of neural cuff electrodes. Multicontact selective neural stimulation has the advantage of activating several muscles via one electrode, and it requires much less energy than epimysial or intramuscular stimulation as thresholds are at least 10 times lower. Human trials have demonstrated the feasibility of this approach for hand movements [18, 21, 22] but, as it combined multisite neuromuscular stimulation on all targeted muscles, it leaded to a high number of implanted electrodes (more than 10) and the associated wires from the forearm to the chest and therefore was from this point of view not more advantageous than the original FreeHand system. The limited efficacy of the nerve stimulation was due to both the limited selectivity of the electrode that was used and the simplicity of the stimulation paradigm, essentially monopolar configurations. Indeed, there were four contacts with a global reference far from the electrode and a single contact among the four was used during stimulation. More complex multicontact electrodes were successfully used in the upper limb of humans, i.e., the FINE [23, 24] and the TIME [25, 26]. However, the stimulation paradigm remained limited to monopolar stimulation for which a single active contact was used toward a global ground. This approach was also not the most selective. Moreover, TIME was used for sensory feedback recovery, the FINE and the 4-contact electrode showed limited selectivity, which meant that a high number of implanted elements were needed to target all the required muscles.

Our first hypothesis was that multicontact electrodes implanted above the nerve bifurcations would enable the selective activation of several fascicles from the same nerve. Indeed, upper limb fascicles tend to anastomose and separate over a large part of their length but are somatotopically organized distally from the spinal cord [27]. This selective activation might potentially activate various functions or muscles independently. Thus, depending on the subject, it might be possible to selectively activate agonist groups of muscles to obtain functional outcomes.

Second, we hypothesized that using only two multicontact cuff electrodes around the radial and median nerves in association with optimal current spreading to all the contacts would result in much higher selectivity and thus isolated or functional movements [28]. This approach aims at replacing the use of a single individual cuff with monopolar-like stimulation paradigms or / one muscular electrode per targeted muscle, by multi-contact neural electrodes implanted proximally with 3D current shaping to achieve a high precision in activating the targeted zone within the nerve linked to functional desired movements.

To validate both hypotheses, we carried out intraoperative trials on either the median or radial nerve in eight subjects with tetraplegia during already-scheduled surgeries. This paper presents the very encouraging and innovative results of the study that open the path to the full restoration of hand movements with a minimally invasive medical device.

## Methods

### Subject recruitment and surgery

Eight subjects (male, 35.5 ± 12.2 years old) with C5 complete motor cervical injury (AIS A except P8: AIS B) were included in the protocol (Table 1). The protocol was approved by the ethics committee (CPP Sud Méditerranée, #ID-RCB:2014-A01752–45) and the clinical trial (Reg. Number #NCT03721861) was performed in accordance with the Declaration of Helsinki.

**Table 1:** Subject characteristics

Subject	Age (years)	Interval time since the onset of SCI (months)	Nerve stimulated	Giens^**a**^ score
**P1**	23	22	left radial	0
**P2**	25	34	left radial	1
P3*	31	21	left radial	2
**P4**	32	29	left radial	2
**P5**	54	7	right median	2
P6*	32	33	right median	1
**P7**	55	22	left median	2
**P8**	32	192	right median	1

*Subjects P3 and P6 are the same individual who underwent two surgeries

^a^The Giens classification is used to list active muscles below the elbow (allowing an active movement against gravity and resistance) in tetraplegic patients

Inclusion criteria were complete motor tetraplegia, age between 18 and 65 years old, neurological stability for at least 6 months, programmed surgery to restore elbow extension, and positive electrical mapping for at least one flexor or one extensor.

**Fig. 1:**
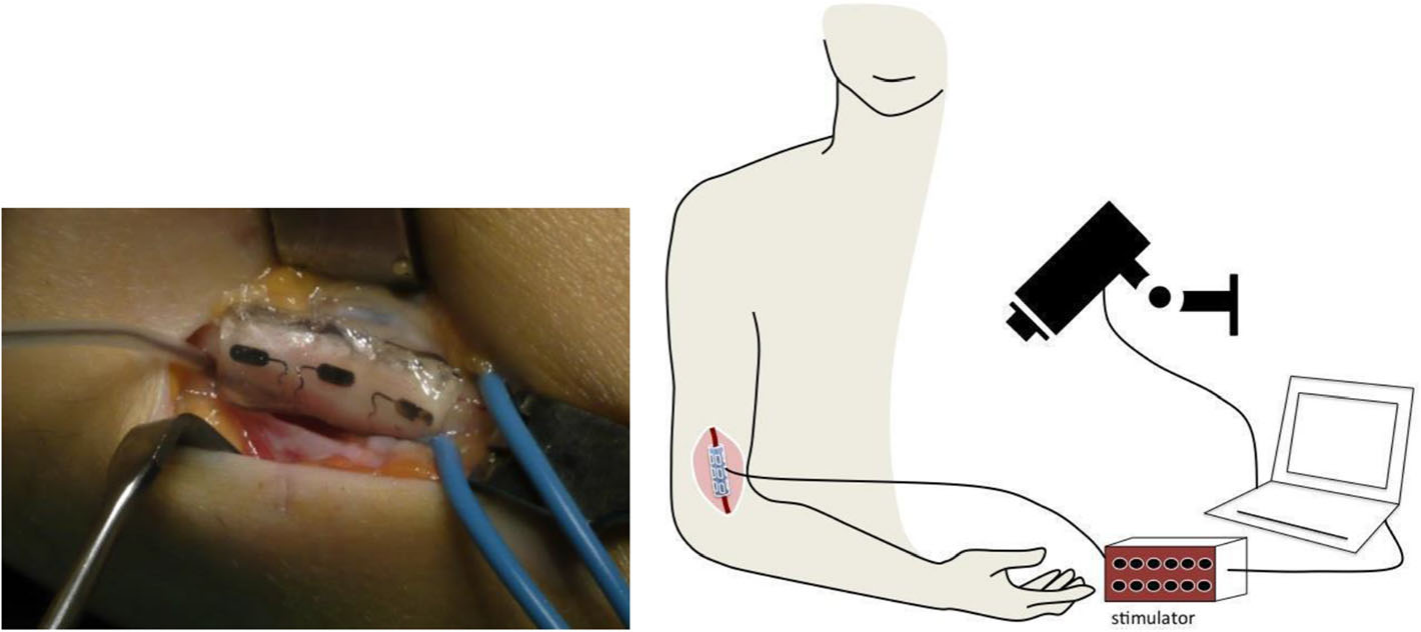
Left: cuff electrode wrapped around median nerve in P6. Right; Setup description: a multicontact cuff electrode is placed around the median or radial nerve and connected to an electrical stimulator. A synchronized video recording enabled post-processing

Each participant gave written informed consent. For ethical reasons, the clinical trial was conducted during a scheduled surgery − a tendon transfer to recover active elbow extension – in order to avoid a dedicated surgery. Moreover, the time slot was 30 min to keep the total time under anesthesia below 2 h. Thus, a single nerve, the median or radial nerve, was chosen depending on the surgical approach and intraoperatively tested on each subject. In the first visit, we performed a mapping of the targeted muscle groups, namely thumb, fingers and wrist in flexion/extension, and this along with other inclusion criteria determined whether a patient would be included. After exposure of the nerve about 5 cm above the elbow, one cuff electrode was placed around the targeted nerve and then gently sutured to avoid its displacement during the trial (Fig. 1).

The radial nerve provides motor innervation to muscles in the arm and forearm that are mostly extensors [29, 30]: brachialis, brachioradialis (BR), extensor carpi radialis longus (ECRL), supinator, extensor carpi radialis brevis (ECRB), extensor digitorum communis (EDC), extensor carpi ulnaris (ECU), extensor digiti quinti (EDQ), abductor pollicis longus (APL), extensor pollicis longus (EPL), extensor pollicis brevis (EPB), and extensor indicis proprius (EIP).

Finger extension is ensured by the contraction of EDQ, EDC and EIP. Wrist extension is ensured by the contraction of the ECRL, ECRB and ECU muscles. Thumb extension involves EPL and EPB. Forearm supination is provided by the contraction of the supinator muscles.

The median nerve innervates predominantly the flexor muscles of the forearm and the hand. Wrist flexion is ensured by the flexor carpi radialis (FCR) and the palmaris longus (PL, not always present). Pronator teres (PT) and pronator quadratus (PQ) provide pronation of the forearm and the wrist, respectively. Digit (except thumb) flexion is ensured by the flexor digitorum superficialis (FDS), the flexor digitorum profundus (FDP, 2nd and 3rd fingers) and the lumbricals (L, 1st and 2nd fingers). Thumb flexion is ensured by the flexor pollicis longus (FPL) and flexor pollicis brevis (FPB) also innervated by the ulnar nerve). Thumb abduction and thumb opposition are ensured by the abductor pollicis brevis (APB) and opponens pollicis brevis (OPB), respectively.

The ulnar nerve was not considered in this study even though it would have provided access to muscles inducing additional flexions (4th and 5th fingers and flexor carpi ulnaris in particular), but the aim of the study was to investigate a minimally invasive solution for grasping.

### Electrodes

A 4-mm diameter, 2-cm length cuff electrode was used for radial nerves (3 × 3 contacts, Cortec GmbH, Freiburg, Germany) and a 6-mm diameter, 2-cm length cuff electrode was used for median nerves (3 × 4 contacts, Cortec GmbH, Freiburg, Germany, Fig. 1). The cuff electrode contacts (2.79 × 0.79 mm2, 5.9-mm spacing between two longitudinal adjacent contacts) were 90/10 Pt/Ir made and embedded with silicone (Nusil). Electrode integrity was checked before and after surgery by a continuity test in saline solution.

### Stimulation protocol

The stimulator was co-developed by Axonic and the University of Montpellier based on the architecture described in [31]. The stimulator can distribute the current over the 12 contacts with a ratio between 1/15 to 15/15 of the total injected current. This makes it possible to drive independently and in synchrony the 9 or 12 contacts of the cuff electrode, which can be further configured as anode or cathode during the active phase of the stimulus. The intensity (up to 2.4 mA, 8-bit resolution), pulse width (up to 511 μs, step 2 μs) and frequency (up to 50 Hz) are programmable and the compliance voltage is 16 V. The stimulator follows the essential safety requirements concerning both the embedded software and hardware. The stimulator is fully insulated from the control PC. The waveform stimulation is biphasic, asymmetric and charge balanced, and a delay of 100 μs between the active and recovery phases is inserted [32]. To assess the selectivity of the multicontact cuff, we selected up to 35 configurations of stimulation (Tables S3-S4, Fig. S5, Additional file 1) based on the simulation study and validated in preclinical studies [28]. We showed that they provide a panel of optimal current spreading over the 12 contacts that induces selective fascicular activation. The stimulation testing is composed of a first scale adjustment of the intensity using a classic tripolar ring configuration: the threshold value that induces a visible movement is determined. Then, automatic scanning is programmed from approximately 80 to 250% of this threshold value (Table S6, Additional file 1). The pulse width and frequency are fixed to 25 Hz and 250 μs for all trials, and the configurations and intensities are scanned every 2 s (1 s ON-1 s OFF) to limit fatigue.

### Outcome assessment

During the scan, the surgeon labeled the movement and evaluated its grade on the MRC (Medical Research Council) scale. A double check was performed off-line with the synchronous audio/video recordings (LabChart software, ADinstrument, Austria) by a second independent physician. The video captured wrist and hand movements (supplementary material).

The muscle response to electrostimulation was characterized by extrapolation to the MRC score (Table S7, Additional file 1). First, muscle responses were characterized intraoperatively by the orthopedics surgeon. A second analysis using videos was carried out postoperatively with a physical medicine and rehabilitation (PM&R) physician. As the muscle response was induced by electrostimulation, no contractions were assessed higher than 4 on the MRC scale. The movement was evaluated rather than the exerted force, and thus a complete movement against gravity was rated MRC < 4 regardless of the force produced. Similarly, a movement initiated but incomplete in terms of trajectory was rated conservatively between 1 and 2. Last, a contraction obtained without any movement was rated as 1.

As the experiment was included in an already scheduled surgery, we were limited to use surface EMG recorded intra-operatively with surface sterile patches 2”× 2” (TENSproducts, USA) placed over the forearm. The surgeon positioned 2 pairs of electrodes over FDS-FDP, FPL. The reference electrode was placed on the shoulder. Acquisition was synchronized with the stimulator through a GTec amplifier with LabChart software (ADinstrument, Austria): notch filter 50 Hz, band pass Bessel 2nd order 0.5 Hz − 1 kHz, Amplification 1000, sampled at 10 kHz, 16 bits. The most important movements to be quantitatively assessed to study grasping were the fingers and thumb flexion. Data were post processed to assess the M-wave level of energy for 2 muscles’ group (FDS-FDP, FPL). Depending on the subject, the channel with the highest signal-to-noise ratio was selected for each muscle. Moreover, all data contaminated by movement artifacts (mainly due to strong wrist movement) were discarded. EMG signal was segmented into 40 ms sets (400 points) between 2 stimulation pulses. Finally, as surface EMG may embed signals from several muscles, we further discriminate signals from the 2 studied muscles’ group using a continuous Meyer wavelets analysis. We then defined the time frequency domains for each studied muscles and compute the Root Mean Square (RMS) energy within this area to extract the recruitment curve. The recruitment curves were normalized to the maximum RMS obtained for each muscle and each patient over the whole session. Reader can refer to attached supplementary materials for details.

### Indexes

Data analysis was performed off-line using MATLAB (Mathworks) for the participants implanted on the median nerve only (P5 to P8). We present data relative to the thumb and fingers flexions as they were observed in all the four participants, and are two important movements needed for grasping function. RMS values of compound muscular action potential (CMAP) were normalized to the maximum RMS value of CMAP of the movement to express the response as a fraction of the full movement activation (recruitment *r*). For each stimulation configuration (*conf*), cathode conformation (*cath*) and stimulation intensity (*I*), the selectivity index (*SI*) was calculated as the recruitment *r*_*conf,cath,m*_ of the considered muscle (*m*) divided by the sum of the recruitment of the 2 movements studied (thumb flexion and fingers flexion) as follows:

$$ {SI}_{conf, cath,m}=\frac{r_{conf, cath,m}(I)}{\sum_{j=1}^2{r}_{conf, cath,j}(I)} $$

A selective and functional criterion, the SIR, was defined based on [33, 34] The SIR is true if, for a single muscle *m*, a given configuration *conf* and a conformation *cath*, the SI index and the recruitment r are above 60%.

$$ SIR= true\ if\ {SI}_{conf, cath,m}>60\%\&{r}_{conf, cath,m}>60\% $$

## Statistics

A Pearson’s Chi-squared test was performed for the configurations, which reached the SIR to assess the independence between the configurations, the movements and the participants.

## Results

The eight participants presented tetraplegia and were candidates for tendon transfer surgery. Depending on the surgery, either the radial (subjects P1 to P4) or median (subjects P5 to P8) nerve could easily be exposed and stimulated. During surgery, a limited time window of about 30 min was dedicated to the entire experimental procedure, including cuff electrode placement around the nerve. The objective was to avoid increasing the duration of general anesthesia and prolonged lying. Thus, the 3D-shaping current configurations were pre-computed and only the scale factor of the current was adjusted for each subject individually based on threshold detection of muscle contraction through a classical tripolar configuration. Indeed, in a theoretical study combined with a preclinical trial we showed that several 3D-shapings were optimal, depending on the size and location of the targeted area within the nerve, and this was almost independent of the subject, except the global scale [28]. A 3-ring composed of three contacts was used for the radial nerve and a 3-ring of four contacts was used for the median nerve as its diameter was larger and the required selectivity was higher. During surgery, we determined the current intensity threshold for each subject and then the predetermined configurations were automatically scanned with increasing intensities following pre-computed and subject-specific ranges. Both a physician and a trained engineer systematically analyzed all videos and extracted individual forearm movements described in the following.

### Induced movements

During radial stimulation, wrist dorsiflexion and finger extension were obtained in all four subjects (P1 to P4). Thumb extension was also obtained in subjects P1 and P3, and forearm supination and elbow flexion were obtained in subjects P2 and P4. During median stimulation, wrist palmar flexion and finger and thumb flexion were obtained in all four subjects (P5 to P8). Thumb opposition was also obtained in subjects P5, P7 and P8 and forearm pronation in subjects P6, P7 and P8.

### Isolated movements

**Fig. 2:**
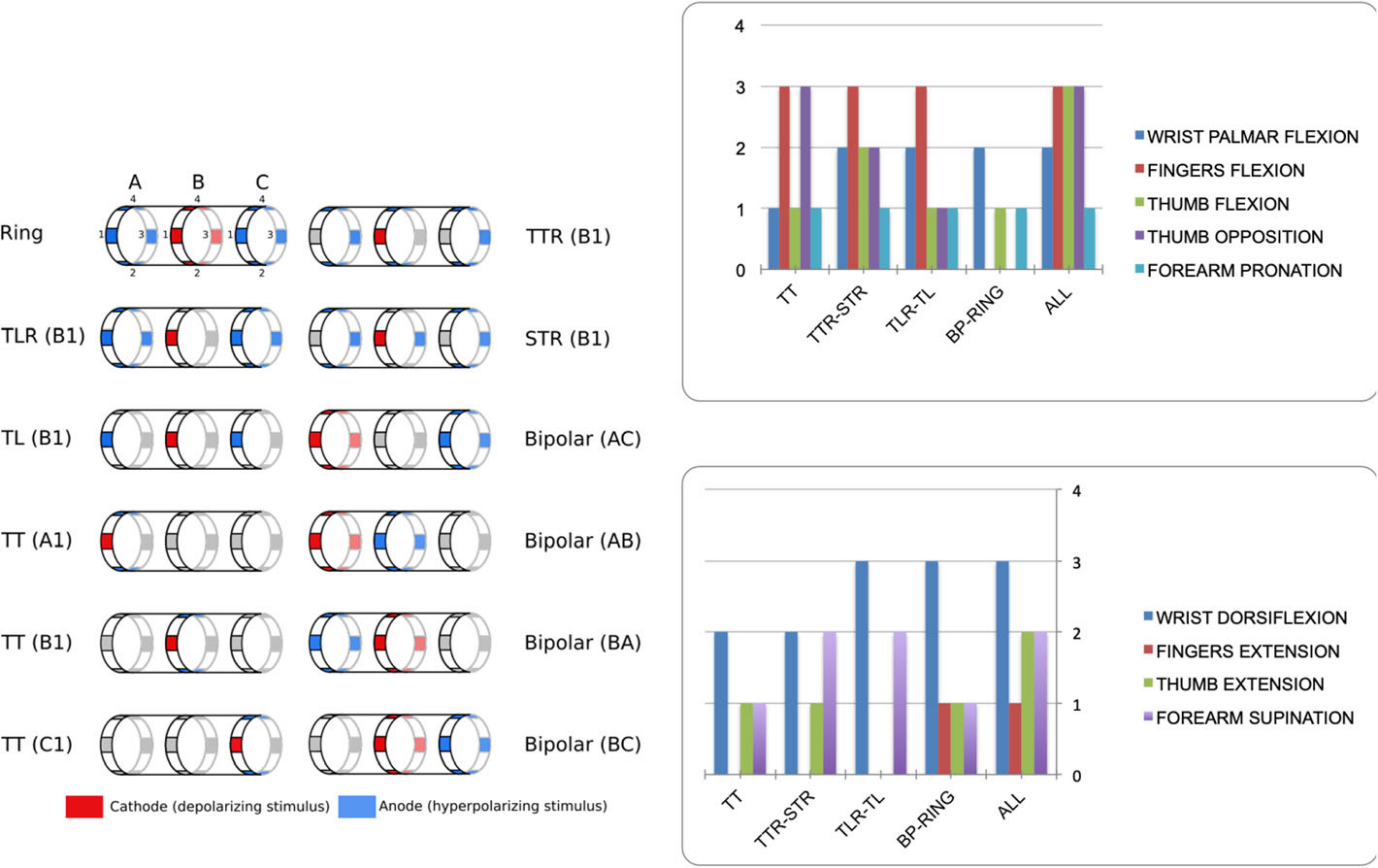
Left: Electrode configurations - Right: Number of subjects who produced an isolated movement with each electrode configuration (sorted from the most to the least selective). Top: Median nerve. Bottom: Radial nerve

With some of the electrode configurations, isolated movements could be produced at low intensities (Fig. 2). As current intensity increased, the amplitude of the movement increased and compound movements progressively appeared: the activated area under the active electrode contact progressively enlarges with dynamics depending on the configuration. The theoretical study predicted that the most selective configuration (the smallest activated area) was the tripolar transverse (TT), then the tripolar transverse+external ring (TTR) and steering+external ring (STR), then the tripolar longitudinal with or without external rings (TLR, TL), and finally the classical bipolar (BP) and tripolar (Ring) configurations. Figure 2 shows the number of subjects who were able to achieve isolated movements in function of the configurations tested. Concerning radial stimulation, TLR-TL and Ring were the most efficient configurations to achieve selectivity of wrist dorsiflexion (3 out of 4 subjects). Forearm supination was achieved for 2 out of 4 subjects using TTR-STR and TLR-TL. Thumb extension was reached in 1 out of 4 subjects using TT, TTR-STR and BP-Ring. Finger extension was observed for only one subject using the BP-Ring configuration. Results for median stimulation show that the observed isolated movements were obtained using TTR-STR. Isolated fingers flexion was obtained for 3 out of 4 subjects using the TTR-STR, TT or TLR-TL configurations. Selective activation of thumb opposition was reached for 3 out of 4 subjects using the TT configuration. Ring could not achieve selective thumb flexion or opposition. Details of the used configuration are shown on Fig. 2.

### Combined movements

For several electrode configurations, combined movements were elicited for both radial and median nerve stimulation (Table 2). For some configurations, one isolated movement was elicited for lower current intensities and novel concomitant movements appeared with increasing intensity.

**Table 2:** Compound movements obtained with different configurations

**Radial nerve - compound movements**							
**Subject**	**Fingers+thumb extension**	**Fingers+wrist extension**	**Wrist+thumb extension**	**Wrist+thumb+fingers extension**			
**P1**	TT(C1)	*Not observed*	*Not observed*	TT(A2)			
	TTR(B2)			TTR(B2)			
	TLR(B2) TL(B2)			TLR(B2) TL(B2)			
	Ring BP(AB)			BP(AB;AC)			
**P2**	*Not observed*	-	*Not observed*	*Not observed*			
		TTR(B1)					
		TL(B1)					
		-					
**P3**	*Not observed*	TT(B1)	TT(B1;C1;C2)	TT(A1;C1;C3;B1)			
		TTR(B1)	-	-			
		TLR(B1;B2;B3) TL(B1)	TL(B3)	TL(B1;B2;B3)			
		BP(BC;CB;AB;AC)	-	BP(BC;CB;AB;AC) Ring			
**P4**	*Not observed*	TT(B1;B2)	*Not observed*	*Not observed*			
		-					
		TLR(B1;B2;B3) TL(B1)					
		BP(AB)					
**Median nerve - compound movements**							
**Subject**	**Fingers + thumb flexion**	**Fingers flexion + thumb opposition**	**Wrist flexion + thumb opposition**	**Wrist flexion + thumb flexion**	**Fingers + thumb flexion + thumb opposition**	**Fingers + wrist flexion**	**Wrist + thumb + fingers flexion**
**P5**	TT(A1;B1;B3;B4;C3)	TT (A4)	*Not observed*	-	*Not observed*	*Not observed*	-
	TTR(B2,B3) STR(B1;B2;B3)	TTR (B4)		-			TTR(B1;B3)
	TL(B3;B4) TLR(B1,B2,B4)	-		TLR(B3)			TLR(B1;B2;B3) TL(B1;B2)
	BP(BC,AB,BA,AC) Ring	-		-			Ring
**P6**	TT(A1;A4;B1)	*Not observed*	*Not observed*	*Not observed*	*Not observed*	*Not observed*	TT(A4)
	TTR(B1;B4) STR(B1)						TTR(B1) STR(B1)
	TLR(B1;B4) TL(B1)						TL(B1) TLR(B1;B4)
	Ring BP(BA)						-
**P7**	TT(A3;A4;B3;B4;C3)	TT(A1;B1;B2;C1)	*Not observed*	*Not observed*	TT(A1)	-	-
	TTR(B3)	TTR(B2) STR(B1;B2)			-	-	TTR(B3)
	TLR(B1;B2;B3) TL(B2;B3)	TL(B1)			TLR(B1)	-	TL(B3)
	BP(BC;AB;BA;AC) Ring	-			-	Ring	-
**P8**	-	*Not observed*	TT(B3)	-	*Not observed*	-	TT(A2;B2)
	TTR(B2)		-	STR(B2)		-	TTR(B2) STR(B2)
	-		TL(B3)	-		TLR(B4)	TLR(B2;B3)
	-		-	-		Ring	-

Concerning the radial nerve stimulation, useful combined movements to open the hand (fingers+thumb extension or fingers+thumb+wrist extension) can be obtained for all subjects except P2; indeed, Table 2, Fig. S1-Table S2 (Additional file 1) show that thumb extension was never achieve for this patient so an incomplete opening is occurring. For the median nerve stimulation, the most important result is that we obtain fingers +thumb flexion for all subjects so a key grip is possible even though not finely controlled as both flexions occur simultaneously. Considering thumb opposition, some power grip is possible for P5 and P7. In both cases, as selectivity is lower than for isolated movements, an increased number of configurations leads to the same functional result. Concerning the obtained functional movements, as muscles are contracted simultaneously, the quality of the movement is not accurately controlled. Table 2 reports other, almost unwanted, composed movements that will be avoided.

### Semi-quantitative assessment of movement

In intraoperative conditions and with a limited time window, we assessed movement with a modified semi-quantitative scale (modified Medical Research Council: MRC) to estimate the quality of the movements. The scale indicated that large and strong movements were achieved in all the subjects without any prior muscle reinforcement. We examined the MRC only for median nerve stimulation-induced movements, as we limited the reporting to opening/extension capabilities for the radial nerve stimulation. As observed for the median nerve stimulation, several configurations induced similar movements; Table 3 presents the most efficient one.

**Table 3:** Highest strength obtained for each movement using an adapted Medical Research Council (MRC) scale. This table presents the highest MRC grades obtained. The range of current is the one that enables recruitment modulation (0–100%). These configurations reach the maximum recruitment i.e. 100% based on EMG recordings (fingers and thumb flexions only) and MRC scaling (all)

***Median nerve***					
Patient	Wrist flexion	Fingers flexion	Thumb flexion	Thumb opposition	Forearm pronation
**P5**	**MRC < 3** **680** μA**–1640** μA TL(B1)	**MRC < 3** **800 μA–1640 μA** BP(AC)	**MRC < 2** **1440 μA–1920 μA** TT(B4)	**MRC < 2** **680 μA–1640 μA** TTR(B4)	*Not Obtained*
**P6**	**MRC < 4** **400 μA–720 μA** TL(B1)	**MRC < 4** **400 μA–720 μA** TL(B1)	**MRC < 4** **480 μA–720 μA** TL(B1)	*Not Obtained*	**MRC < 4** **747 μA–1120 μA** TT(A1)
**P7**	**MRC < 4** **40 μA–400 μA** Ring	**MRC < 4** **240 μA–400 μA** TL(B1)	**MRC < 4** **240 μA–400 μA** TL(B3)	**MRC < 4** **93 μA- 700 μA** TT(A1)	**MRC < 4** **40 μA–400 μA** TTR(B2)
**P8**	**MRC < 4** **560 μA–933 μA** TT(A4)	**MRC < 4** **240 μA–400 μA** TLR(B2)	**MRC < 4** **240 μA–400 μA** STR(B2)	**MRC < 3** **240 μA–400 μA** TL(B3)	**MRC < 4** **240 μA–400 μA** TL(B4)

This table shows that strong contraction of the desired muscles can be obtained but not always isolated. For instance, the highest score for P6 is obtained with a single configuration TL(B1) i.e. with all muscles contracting simultaneously. On the contrary, each movement has its preferred configuration, even though not providing a completely isolated movement, for subject P8.

From the group of patients who received median nerve stimulation EMG data was processed to assess thumb and fingers flexions (Fig. S8, Additional file 1).

**Fig. 3:**
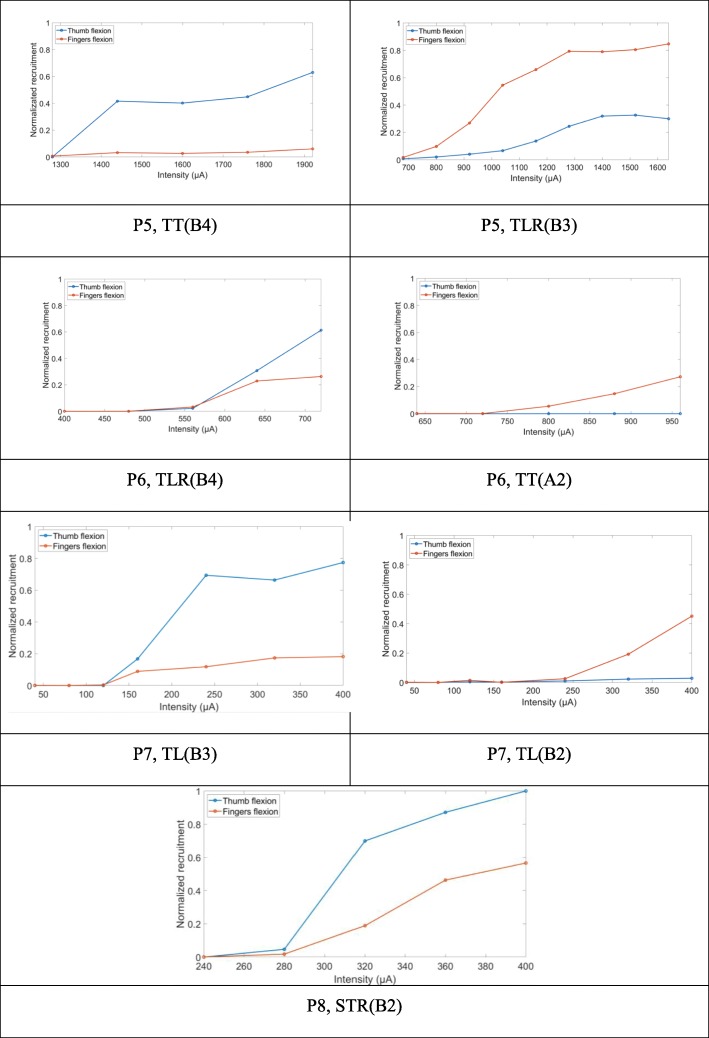
Examples of recruitment curves with selective thumb flexion (left) or fingers flexion (right), or both flexions simultaneously (P8)


The recruitment curves further confirm the video based sorting (Additional file 2). Figure 3 shows the selective activation of either the thumb flexor or the fingers’ flexor, or both in a varying proportion. Figure 3 P5 TLR(B3) illustrates the progressive recruitment of the fingers flexor first and the thumb flexor.

**Additional file 2.** This video shows several video recordings illustrating the obtained movement while stimulating radial or median nerve. In particular isolated movements can be clearly identified while stimulating the same electrode on the median nerve but using different electrode’s configurations.


To further investigate selectivity against configurations and patients, we excluded Ring, Bipolar and TT on A and C rings. Based on the Selectivity Index and the recruitment levels, the SIR criterion was then true for 20 configurations. Among these selective configurations, the statistical tests demonstrated that the configuration is independent of the movement and the participant (respectively *p = 0,61 and p = 0,23)*. This indicates that for each patient, the whole set of configurations should be explored without a priori.

### Toward functional movements

Our ultimate goal was to achieve functional grasping movements. Radial nerve stimulation produced hand opening through the combined activation of thumb, finger and in some cases wrist extension, leading in most cases to a wide opening of the whole hand, which might allow surrounding an object to grasp. We registered several functional grips for median stimulation: key grip, power grip, and hook grip (Table 4): compared to Table 2, the selected grips are limited to the ones with a strong contraction without unwanted pronation or wrist flexion. We were able to obtain at least one type of grip for each subject. It was nevertheless not possible to measure the grip force, and these combined and functional movements were generated with a specific configuration and current intensity level and not by combining isolated movements.

**Table 4:** Functional grasps obtained with configurations*.*

Median nerve - functional movements			
Subject	Key grip	Power grip	Hook grip
**P5**	TT (C3) - TLR(B2;B3;B4) TL (B1*;B2) - *Fingers r = 0.72, Thumb r = 0.41, I = 1400 μA	*Not observed*	*Not observed*
**P6**	TT (A4) - - - **Fingers r = 0.35, Thumb r = 1, I = 960 μA**	- STR (B1*) TLR (B4*) Ring* BP (BA*) *MRC < 4	- TTR (B1;B4) TLR (B1) TL (B3*) - *Fingers r = 0.18, I = 720 μA
**P7**	TT (A1;A4;B4) TTR (B1*;B3) TLR (B1;B3) TL (B1;B3) Ring BP (BC;AB;BA) *Fingers r = 0.68, Thumb r = 0.38, I = 600 μA	- - - BP (AC) **MRC < 4**	TT(B3) - - - **MRC < 4**
**P8**	*Not observed*	TT (A2*,B2*) TTR (B2*) STR (B2*) TLR (B2*;B3*) - *MRC < 4	- - TLR (B4) - **MRC < 2**

*Identifies the highest MRC obtained, and for Key / Hook grip the recruitment of the targeted muscle with the intensity of the stimulation when clean EMG data are available

## Discussion

We implanted a single multicontact cuff electrode around the median or radial nerve in 8 subjects with complete tetraplegia and obtained almost all the movements needed, either combined (Tables 2 and 4) or isolated (Table S2 in Additional file 1, Additional file 2), to restore functional grasping of objects. Indeed, missing key grip for P8 is due to the fact that configuration STR(B2) induced not only a key grip but also some wrist flexion and pronation that could lead to a non-functional movement in the end. For P5 power and hook grips using a combination of fingers flexion TTR(B3) alone or with thumb opposition TTR(B4) was possible but we did not report these movements as functional as the MRC score was lower than 3 and do not provide a powerful grip. Training could probably provide the needed force. Concerning opening the hand, mostly fingers and thumb extension; the movement was obtained with P1. For the others, fingers extension was combined with wrist dorsiflexion – possibly increased by mechanical coupling. For P3, an additional thumb flexion using interleaved TT (C1) can be used to completely open the hand. For P2 and P4, we did not achieve thumb extension probably due to the fact that the cuff has a too limited number of contacts (#3) that should be changed for future trials (Fig. S9, Additional file 1). Indeed, the nerve organization differs from patient to patient and we obviously had not enough contacts within the cuff electrode: on patient P3, at the end of the session, the cuff around the radial nerve was turned about half an inter contact distance and a limited scan to TT configurations was performed. It shows, for instance, isolated fingers flexion that was not observed with the initial position (data excluded as it is a single case without accurate rotation measurement). An augmented number of contacts together with a fine-tuning of all stimulation parameters with some extra configurations tested outside the operating room may greatly extend the number of obtained movements. Finally, all the patients were at the same level of spinal cord injury but not the same Giens score (Table 1) that may lead to muscles that cannot be activated by electrical stimulation.

This approach is the most economical to date in terms of the number of implantable parts, as only two electrodes above the elbow would be needed to restore the main functional movements of the hand for individuals with complete tetraplegia. Besides, on another application, namely lower limb movement restoration on subject with complete paraplegia [35–37], we showed that neural stimulation is more reliable and efficient on a long term (9 years) than epimysial, further confirming the interest of a full neural system [38]. We confirmed the hypothesis derived from theoretical studies that the spatial selectivity obtained with both multicontact electrodes and complex current spreading can provide selective movements. In particular, to our knowledge, we provide for the first time, the evidence that a stimulation of the median nerve can provide a selective activation of the fingers, thumb and wrist flexors. This means that despite anastomosis, which is known to occur in the proximal part of upper limb nerves (in our case above the elbow), fascicularization is functionally relevant even though it differs from individual to individual. We further confirmed, for the first time in human, that complex 3D-current shaping is efficient and that the prior theoretical studies without a priori knowledge of a generic nerve are relevant. Indeed, as demonstrated in our technical paper [28], only the scale factor of the current intensity needs to be adjusted but the exact knowledge of the fascicule organization is not mandatory and in any case is not available in individuals.

These results were obtained with a significant group of eight participants. The next step should be a clinical trial with awake participants. This proof of concept was mandatory from an ethical viewpoint, and it clearly showed the possibility of combining isolated movements through interleaved stimulation paradigms or through deeper investigation of the recruitment obtained with the most relevant configurations, which provide even richer and more complex movements. During the intraoperative testing, automatic scanning showed that recruitment was always progressive for both the amplitude of the movement and the number of primary generated movements. For instance, in subject P7 with configuration TT(A4), we first see the isolated flexion of the fingers with an increasing movement amplitude (233–467 μA), followed by the activation of thumb flexion providing a key grip (> 467 μA), and finally wrist pronation. This illustrates that the selective configurations targeted increasing areas as the intensity increased.

Last, owing to pure neural stimulation, no diffusion to antagonist muscles was observed. The opening function through the stimulation of the radial nerve was more difficult to assess for several reasons. Notably, some subjects were spastic so the hand was almost closed and the stimulation of the radial would have needed more reinforcement and rehabilitation or a surgical elongation of the flexion muscles. But even in this case, we obtained hand opening. Finger extension may induce wrist extension so the co-activation of the median nerve would have been beneficial, but it was not possible in the frame of this trial. However, the system will be able to provide this combined stimulation for future trials.

We did not report the results of the pre-surgical mapping as it was an inclusion criterion for the subjects, but we found that the contraction obtained with surface electrical stimulation through accurate mapping was always less efficient, less selective and unable to activate some of the deep and small muscles. Moreover, we did not stimulate the ulnar nerve so the fifth finger was not activated, but this does not seem necessary to obtain strong grasping.

## Conclusions

The present work is a proof of concept of restoring functional movements through a multi-contact cuff electrodes placed around the median or radial nerve owing to complex current configurations over the 12 contacts. We thus aim at proposing the less invasive solution to date that could restore useful hand grasping for subjects with complete tetraplegia. Our ultimate goal is to offer a device to the patients who cannot benefit from musculotendinous surgery to help them recover grasp movements. We hope to do so using this technology in combination with EMG from sublesional muscles or movements [39] to control nerve stimulation. The next step will be to implant two neural cuff electrodes and let patients control the device themselves through the EMG-based interface.

## Supplementary information

**Additional file 1.** This file includes 9 Tables / Figures numbered S1 to S9 that give further details on obtained isolated movements (Figure S1, Table S2), electrodes’ configurations (Tables S3-S4, Figure S5), the ranges of scanned current amplitudes (Table S6), the modified MRC scale (Table S7), EMG processing details (Figure S8) and a possible fascicular organization (Figure S9).

## Data Availability

Data are essentially medical data that are not publically available.

## Abbreviations

SCI: Spinal Cord Injury
FES: Functional Electrical Stimulation
AIS: Association Impairment Scale
BR: Brachioradialis
ECRL: Extensor carpi radialis longus
ECRB: Extensor carpi radialis brevis
EDC: Extensor digitorum communis
ECU: Extensor carpi ulnaris
EDQ: Extensor digiti quinti
APL: Abductor pollicis longus
EPL: Extensor pollicis longus
EPB: Extensor pollicis brevis
EIP: Extensor indicis proprius
MRC: Medical Research Council
TT: Tripolar transverse
TTR: Tripolar transverse+external ring
STR: Steering+external ring
TLR: Tripolar longitudinal+external ring
TL: Tripolar longitudinal
BP: Bipolar
EMG: Electromyogram
CMAP: Compound Muscular Action Potential
RMS: Root Mean Square
FCR: Flexor carpi radialis
PL: Palmaris longus
PT: Pronator teres
PQ: Pronator quadratus
FDS: Flexor digitorum superficialis
FDP: Flexor digitorum profundus
FPL: Flexor pollicis longus
FPB: Flexor pollicis brevis
L: Lumbricals
APB: Abductor pollicis brevis
OPB: Opponens pollicis brevis
