# Supplementary material for: Selective neural electrical stimulation restores hand and forearm movements in individuals with complete tetraplegia
Source: J Neuroeng Rehabil. 2020 May 19;17:66. doi: 10.1186/s12984-020-00676-4 (PMC7236876; doi:10.1186/s12984-020-00676-4)
Supplement: Supplementary file 2 — Additional file 1. This file includes 9 Tables / Figures numbered S1 to S9 that give further details on obtained isolated movements (Figure S1, Table S2), electrodes’ configurations (Tables S3-S4, Figure S5), the ranges of scanned current amplitudes (Table S6), the modified MRC scale (Table S7), EMG processing details (Figure S8) and a possible fascicular organization (Figure S9). [file 12984_2020_676_MOESM1_ESM.docx]

**Selective Neural Electrical Stimulation Restores Hand and Forearm Movements in Individuals with Complete Tetraplegia**

**Authors:** Wafa Tigra, Mélissa Dali, Lucie William, Charles Fattal, Anthony Gélis, Jean-Louis Divoux, Bertrand Coulet, Jacques Teissier, David Guiraud, Christine Azevedo Coste

**Supplementary Materials**

| 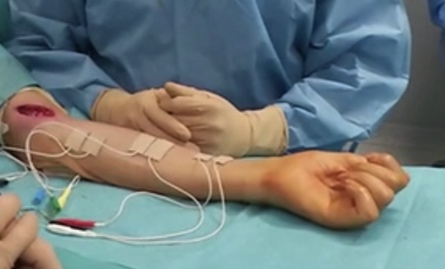 Isolated finger flexion | 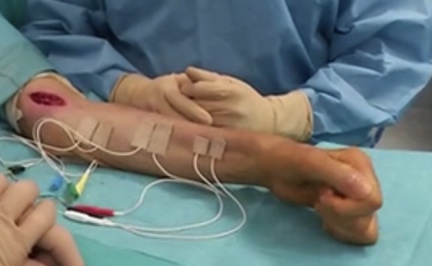Forearm pronation with finger and thumb flexion. | 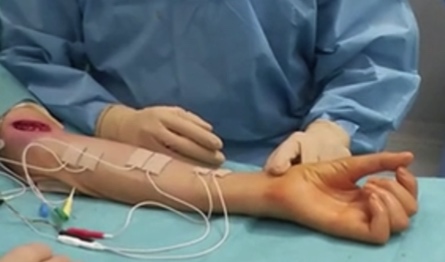Isolated thumb opposition |
| --- | --- | --- |
| 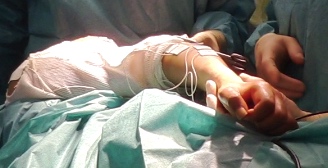 Supination | 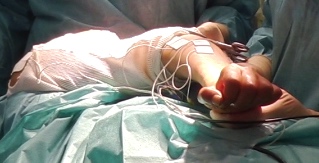Wrist extension | 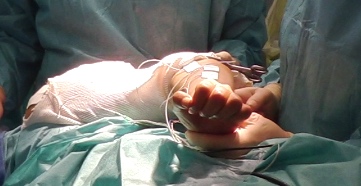 Wrist extension and elbow flexion |

**Fig S1.** Illustration of some of the movements elicited by: (top) median nerve stimulation (P7) (bottom) radial nerve stimulation (P4)

**Table** **S2**. Isolated movements produced according to each electrode configuration. Electrode configurations are organized through 4 clusters of configurations from the most (TT) to the least selective (Ring, BP).

**Radial nerve:**

| **Subject** | **Wrist dorsiflexion** | **Fingers extension** | **Thumb extension** | **Forearm supination** |
| --- | --- | --- | --- | --- |
| **P1** | *Not observed* | Ring | -  TTR(B2)  -  BP(BC) | *Not observed* |
| **P2** | -  TTR(B1)  TL(B1;B2;B3)  Ring BP(BC;AB;AC) | *Not observed* | *Not observed* | -  TTR(B2)  TLR(B2)  - |
| **P3** | TT(A1;B1)  TTR(B1)  TLR(B1;B2;B3) TL(B1;B2;B3)  Ring BP(BC;CB;AB;AC) | *Not observed* | TT(C1;C2;C3) | *Not observed* |
| **P4** | TT(A1;C1)  -  TLR(B3) TL(B3)  Ring BP(AB;AC) | *Not observed* | *Not observed* | TT(B1;B2;B3)  TTR(B1;B2)  TLR(B1;B2) TL(B2)  BP(BC) |

**Median nerve:**

| **Subject** | **Wrist palmar flexion** | **Fingers flexion** | **Thumb flexion** | **Thumb Opposition** | **Forearm pronation** |
| --- | --- | --- | --- | --- | --- |
| **P5** | -  TTR(B1)  TLR(B2; B3) TL(B1;B2)  Ring | TT(A2;B2 )  TTR(B3)  TL(B3)  - | TT(B4)  STR(B4)  TLR(B1)  - | TT(A4)  TTR(B4)  -  - | *Not observed* |
| **P6** | *Not observed* | TT(A2;A3;B4;B3)  TTR(B4) STR(B3)  TLR(B3;B4) TL(B3)  BP(BA) | *Not observed* | *Not observed* | *Not observed* |
| **P7** | *Not observed* | TT(A4;A3;B3;B4;C4;C2)  TTR(B3;B4) STR(B3) TLR(B3) TL(B3)  - | *Not observed* | TT(B1;C1)  STR(B1)  -  - | TT(A2;B2)  TTR(B2) STR(B2;B4)  TLR(B2) TL(B2)  Ring |
| **P8** | TT(A3;A4;B4)  TTR(B2;B4) STR(B3)  TLR(B3;B4) TL(B4)  BP(AB;BA) | *Not observed* | -  STR (B2)  -  - | TT (B3)  -  TL (B3)  - | *Not observed* |

*Electrode configurations*

All configurations were issued from a theoretical study (*27, 32*) in which the optimal current spreading was computed to obtain the best selectivity, efficiency and robustness. Efficiency was defined as the lowest current needed to obtain the best selectivity, and robustness was defined as the range of current that maintained selectivity with a maximum variation of 50%. The wider this range, the more robust the configuration was. Moreover, the initial optimization was based on a fixed target dimension. As the size of fascicules and the associated functional movements were not known, a large set of selective configurations was tested. Based on the size of the activated area, we gathered the seven configuration types into four groups: (i) high selectivity: TT, (ii) mid-selectivity: TTR, STR, (iii) low selectivity: TL, TLR, and (iv) no selectivity: Ring, BP. The term selectivity refers to the targeted zone even though Ring and BP were able to elicit isolated movements. Moreover, the configurations within a group differed in their form, which led to slightly different results.

**Table S3.** Stimulation conﬁgurations for the median nerve. One cathode was activated with a ratio of -12/15 (TT -14/15). Its position was labeled A, B, C (proximal, central, distal ring) and i (1 to 4) for the line, i.e., B1 means the first line of the central ring. The Ring and Bipolar configurations were composed of a full ring as a cathode with 3/15 of the current on each contact. Ratios are in 1/15.

| Configuration name | A1 | A2 | A3 | A4 | B1 | B2 | B3 | B4 | C1 | C2 | C3 | C4 |
| --- | --- | --- | --- | --- | --- | --- | --- | --- | --- | --- | --- | --- |
| Ring | 2 | 1 | 2 | 1 | **-3** | **-3** | **-3** | **-3** | 2 | 1 | 2 | 1 |
| Transverse Tripolar + external Rings (TTR B1) | 0 | 1 | 1 | 1 | **-12** | 3 | 0 | 3 | 0 | 1 | 1 | 1 |
| Tripolar Longitudinal + external Rings (TLR B1) | 1 | 2 | 1 | 2 | **-12** | 0 | 0 | 0 | 1 | 2 | 1 | 2 |
| Tripolar Longitudinal (TL B1) | 6 | 0 | 0 | 0 | **-12** | 0 | 0 | 0 | 6 | 0 | 0 | 0 |
| Bipolar (BP AC) | **-3** | **-3** | **-3** | **-3** | 0 | 0 | 0 | 0 | 3 | 3 | 3 | 3 |
| Steering + external Rings (STR B1) | 0 | 1 | 1 | 1 | **-12** | 0 | 6 | 0 | 0 | 1 | 1 | 1 |
| Transverse Tripolar (TT B1) | 0 | 0 | 0 | 0 | **-14** | 7 | 0 | 7 | 0 | 0 | 0 | 0 |

**Table S4.** Stimulation conﬁgurations for the radial nerve. One cathode is activated with a ratio of -12/15 (TT -14/15). Its position is labeled A, B, C (proximal, central, distal ring) and i (1 to 3) for the line, i.e., B1 means the first line of the central ring. The Ring and Bipolar configurations are composed of a full ring as a cathode with 4/15 of the current on each contact. Ratios are in 1/15. Steering configuration is not possible. Bipolar scan was limited to four out of six possibilities (AC, AB, BC, CB)

| Configuration name | A1 | A2 | A3 | B1 | B2 | B3 | C1 | C2 | C3 |
| --- | --- | --- | --- | --- | --- | --- | --- | --- | --- |
| Ring | 2 | 2 | 2 | **-4** | **-4** | **-4** | 2 | 2 | 2 |
| Transverse tripolar + external rings (TTR B1) | 1 | 1 | 1 | **-12** | 3 | 3 | 1 | 1 | 1 |
| Tripolar longitudinal + external rings (TLR B1) | 2 | 2 | 2 | **-12** | 0 | 0 | 2 | 2 | 2 |
| Tripolar longitudinal (TL B1) | 6 | 0 | 0 | **-12** | 0 | 0 | 6 | 0 | 0 |
| Bipolar (BP AC) | **-4** | **-4** | **-4** | 0 | 0 | 0 | 4 | 4 | 4 |
| Transverse tripolar (TT B1) | 0 | 0 | 0 | **-14** | 7 | 7 | 0 | 0 | 0 |


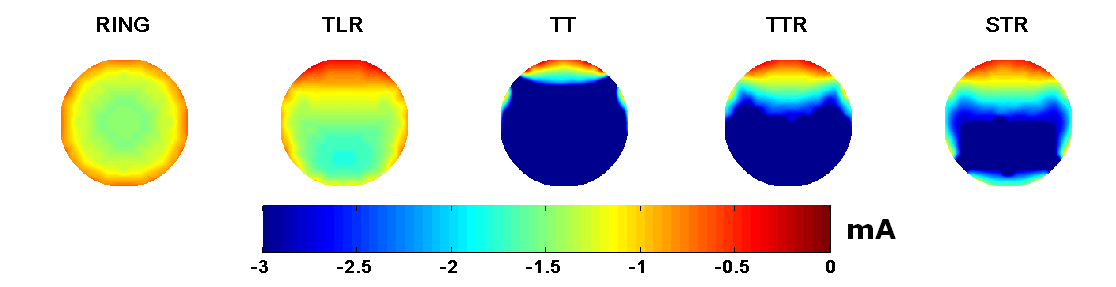


**Fig S5.** Schematic representation of a nerve cross-section. Activation threshold maps were obtained with a modeling study. The sizes of the activated area depend on the configurations *(27).*

*Stimulation scanning parameters*

The current ranges were adjusted for each participant and for the conditions during the surgery, and the set of scanned intensities was therefore specific to each trial.

**Table S6.** Stimulation parameters. For subject P8, the second scan was incomplete due to too-strong contractions for some configurations.

| **Subject** | **Tested intensities (µA)** | **Tested intensities (TT configurations, µA)** |
| --- | --- | --- |
| **P1** | 40 80 120 160 240 320 360 400 | 93 187 280 373 467 560 653 747 |
| **P2** | 160 192 224 256 288 320 352 384 416 448 480 512 544 576 608 640 | Incomplete data |
| **P3** | 400 480 560 640 720 800 880 960 | 1st scan  1773 1960 2147 2240  2nd scan  1027 1213 1400 1587 1773 1960 2147 |
| **P4** | 320 360 400 440 480 520 560 600 640 | 1st scan  560 700 840 980 1120 1260 1400  2nd scan  700 840 980 1120 1260 1400 1540 1680 |
| **P5** | 680 800 920 1040 1160 1280 1400 1520 1640 | 1493 1680 1867 2053 2240 |
| **P6** | 400 480 560 640 720 | 747 840 933 1027 1120 |
| **P7** | 40 80 120 160 240 320 400 | 93 140 187 233 280 373 467 700 |
| **P8** | 1st scan  240 280 320 360  2nd incomplete scan  40 80 104 120 160 200 240 264 288 312 320 336 360 400 440 480 520 560 600 | 560 700 747 793 840 887 933 1027 |

**Table S7.** Adapted MRC scale to take into account the intraoperative conditions as the hand position is constrained by the surgery underway

| MRC=0 | No movement |
| --- | --- |
| MRC<1 | Contraction without movement |
| MRC<2 | Initiated but incomplete movement |
| MRC<3 | Initiated but incomplete movement against gravity |
| MRC<4 | Complete movement against gravity but without force assessment |

*EMG post processing*

The EMG data were first split in sets of 40 ms (400 points) corresponding to the period of stimulation (25 Hz). Then, All bins, except the first one, were averaged for each single trial (i.e. for a given patient, configuration and intensity amplitude). The channels with the highest signal-to-noise ratio were selected to analyze the M-waves of EMG responses of the fingers’ flexors and thumb flexor. As both M-waves are sometimes present in the same channel due to the poor selectivity of surface EMG, we further used Meyer continuous wavelet transform to discriminate between both M-waves in the time and frequency domains. Indeed the areas in the time domain overlap and the FFT was not able to discriminate. The time-frequency domains were determined: for FDS-FDP (6-24.9 ms, 33-210 Hz) and for FPL (0.3-16.9 ms, 247-495 Hz). These domains were the same for the 4 patients (P5 to P8 - median nerve surgery) leading to a common time-frequency signature of a specific muscle over the 4 individuals. Figure S8 illustrates the processing. Interestingly, time-frequency zones were the same for all the 4 patients (1 out of 4 has only EMG for fingers flexion). Shorter and faster M-waves for thumb flexion than fingers’ flexion were observed. The time-frequency areas were selected so that they are not overlapped even though they did not cover the whole time-frequency content of each M-wave. Indeed, the RMS computation is still valid as we compare normalized values.

**
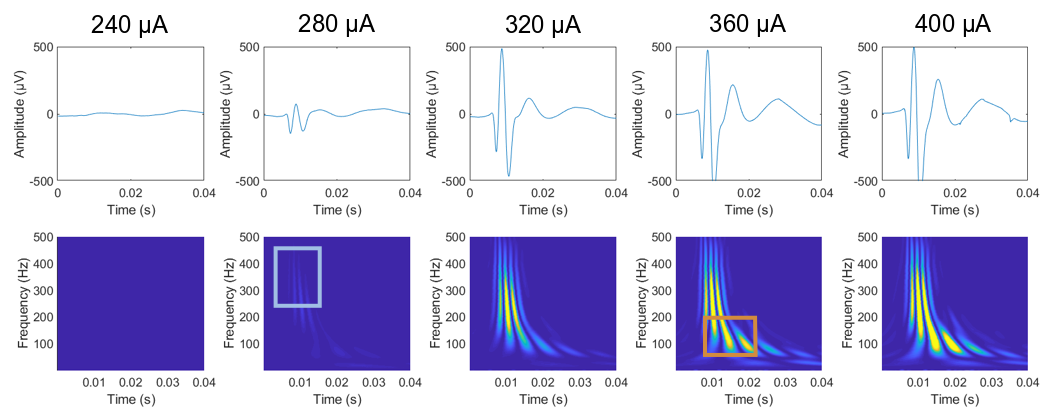
**

**Fig. S8. Top**: example of mixed M-waves on patient P8 for five different amplitudes of current. Bottom: corresponding wavelet transform with the 2 areas identified as specific to thumb flexion (blue rectangle) and fingers’ flexion (orange rectangle) fully correlated to MRC video based quotation.

The recruitment curves are then computed as the RMS value of each defined domain normalized by the maximum RMS value for a each patient, each zone. From this, selectivity and SIR can be computed.

*Discussion on contact selection*

Considering the contact that elicits the contraction of the targeted muscle on one hand, and the used configuration on the other hand, we can have an idea of the location and the size of the fascicules. Indeed, the fascicle should be below or in between contacts that elicits a contraction, furthermore, the very selective configurations TT have a small area of possible activation contrary to TL-TLR that can drive large areas. Combining both gives a rough idea of the size and location as shown on figure S9.


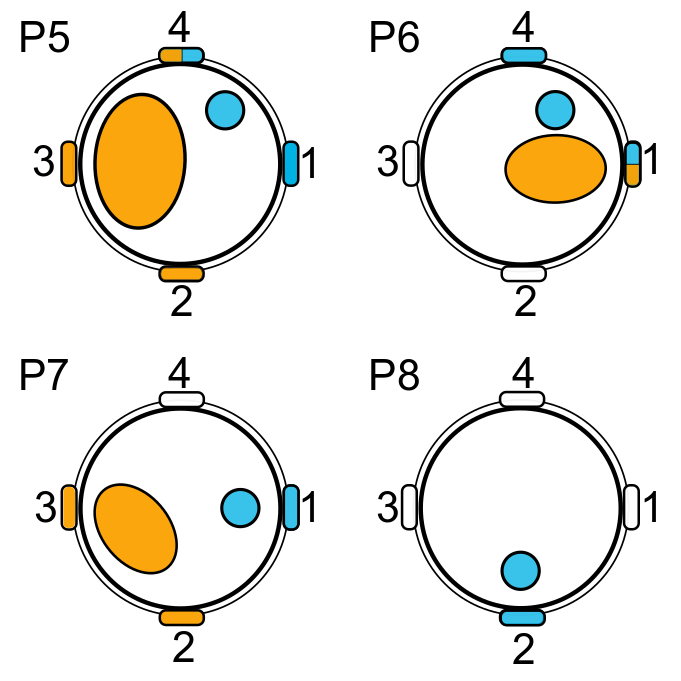


**Fig. S9:** blue contacts elicit thumb flexion while orange ones elicits fingers flexion. The fact that Fingers flexion can be activated by 3 contacts (P5) may be due to a large fascicle while the fact that most of thumb flexion is elicited with only one contact (P7-P8) and never by 3, is in favor of a smaller fascicle. More selective configurations are effective when the fascicule is supposed to be below the contact (see Figure S5).
